# Supplementary material for: Splice-Junction-Based Mapping of Alternative Isoforms in the Human Proteome
Source: Cell Rep. Author manuscript; Available in PMC 2020 Jan 15. (PMC6961840; doi:10.1016/j.celrep.2019.11.026)
Supplement: 3 [file NIHMS1546469-supplement-3.zip › DF2/PXD0010154/Liver-378-Q8IWW6-HQDTDQEKYGLLNVTK.pdf]

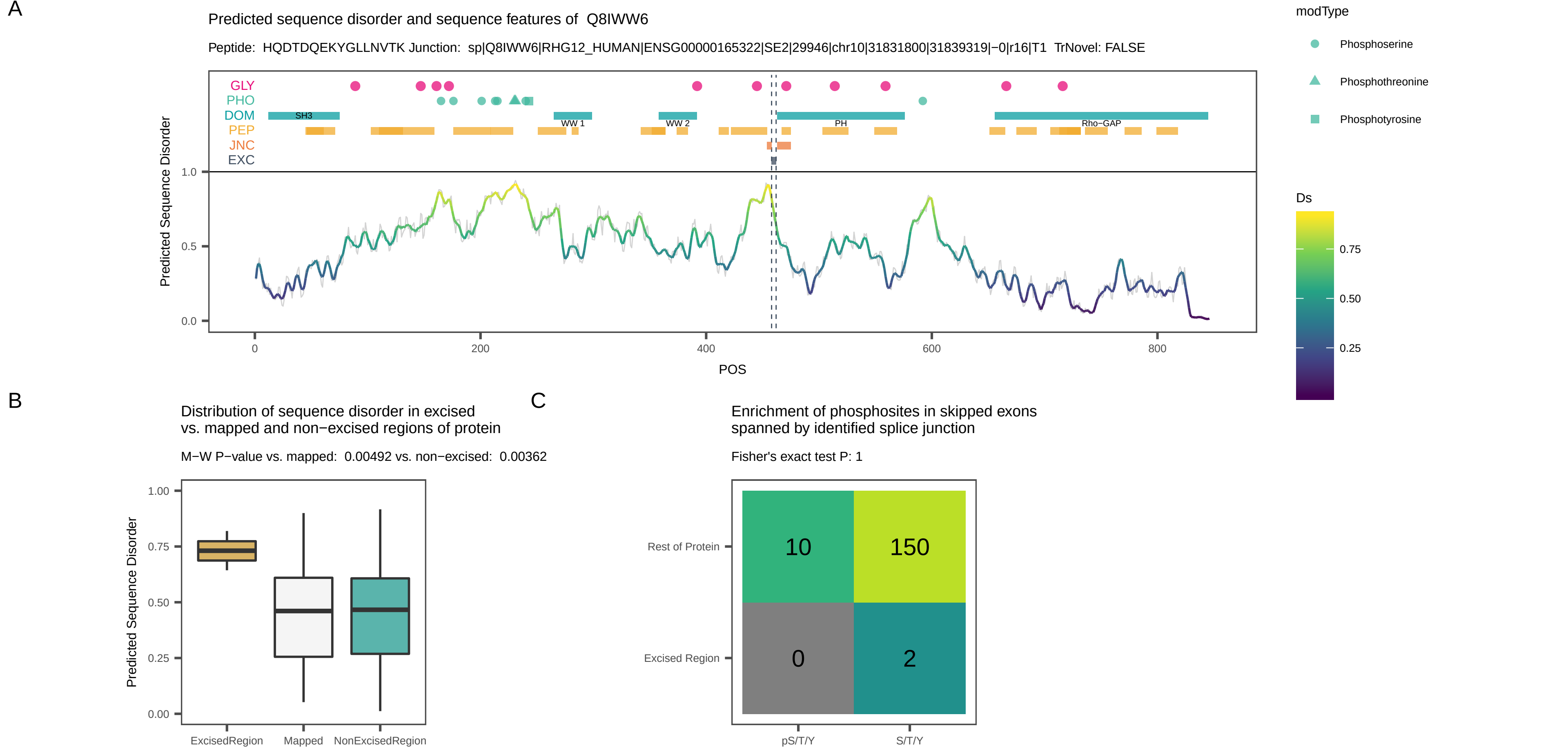
A
Predicted sequence disorder and sequence features of Q8IWW6
Peptide: HQDTDQEKYGLLNVTK Junction: sp|Q8IWW6|RHG12_HUMAN|ENSG00000165322|SE2|29946|chr10|31831800|31839319|−0|r16|T1 TrNovel: FALSE
GLY
PHO
DOM
PEP
JNC
EXC
SH3
WW 1
WW 2
PH
Rho−GAP
Predicted Sequence Disorder
1.0
0.5
0.0
0
200
400
600
800
POS
modType
Phosphoserine
Phosphothreonine
Phosphotyrosine
Ds
0.75
0.50
0.25
B
Distribution of sequence disorder in excised vs. mapped and non−excised regions of protein
M−W P−value vs. mapped: 0.00492 vs. non−excised: 0.00362
Predicted Sequence Disorder
1.00
0.75
0.50
0.25
0.00
ExcisedRegion
Mapped
NonExcisedRegion
C
Enrichment of phosphosites in skipped exons spanned by identified splice junction
Fisher's exact test P: 1
Rest of Protein
Excised Region
10
150
0
2
pS/T/Y
S/T/Y
